
JOURNAL INFORMATION
==============================
NLM Title Abbreviation: Buffalo Med J Mon Rev Med Surg Sci
NLM Journal ID: 0004276

The Buffalo Medical Journal and Monthly Review of Medical and Surgical Science

Publisher: Biomedical Journal Digitization Project

ARTICLE INFORMATION
==============================
PMCID: PMC8932447
PMID: 35376944
Article version: 1
Subjects: Editorial Department

The North American Medical Reporter

Print publication date: 1858 Dec
Volume: 14
Issue: 7
First page: 447
Last page: 447
License: This work is free of known copyright restrictions. For more information, please see PMC Back Issue Digitization.
License URL: https://creativecommons.org/publicdomain/mark/1.0/

==============================

safer and less hazardous to life than the most of preparations used for this purpose, and by just such quacks as he.

Yours, &c., G. H. C.

---

*The North American Medical Reporter.* Edited by W. Elmer, M. D.

This is the title of a new journal published by W. A. Townsend & Co., New York, the publishers of Braithwaite's Retrospect. It is to be regretted that a journal which is evidently designed to be issued in a kind of connection with such a work as Braithwaite's should not take a more dignified position than the one before us. It is designed to present an index of the interesting articles which appear in the various periodicals to which it has access; so far it is good; but in addition, even the first number, which it would be policy to keep free from every thing objectionable, we regret to say, contains many things which the regular profession cannot but condemn. We should not have mentioned this publication, had it appeared under the guise of an eclectic or homeopathic journal, but the position which it aspires to assume, the circulation which it hopes to obtain among the regular profession, and especially its apparent connection with the reprint of Braithwaite's Retrospect, make it necessary for us to speak of its claims on the profession. A journal which contains a favorable notice of "The American Family Physician or Domestic Guide to Health," by John King, M. D., Professor of Obstetrics and Diseases of Women and Children in the *Eclectic* College of Medicine, Cincinnati; and the "Pronouncing Medical Lexicon," by C. H. Cleveland, M. D., Professor in the same institution, can not demand our support or sanction. We say nothing of an editorial article which reads as though it had been copied from the advertising sheet of a daily paper, announcing a new hair invigorator, the formula "to be found in a subsequent part of this number, which constitutes one of the best if not the only *rational* preparation for restoring the hair to its original or healthful condition." These are undignified and professional, and we cannot commend a publication which, like this, either panders to the interests of an eclectic, or the discoverer of a hair wash, or puts himself on a level with them, and indorses their opinions.
